# Network inference through synergistic subnetwork evolution

**DOI:** 10.1186/s13637-015-0027-4

**Published:** 2015-11-27

**Authors:** Lipi Acharya, Robert Reynolds, Dongxiao Zhu

**Affiliations:** 1 Dow AgroSciences, 9330 Zionsville Road, Indianapolis, IN 46268 USA; 2 Department of Computer Science, Wayne State University, 5057 Woodward Avenue, Detroit, MI 48202 USA

## Abstract

Study of signaling networks is important for a better understanding of cell behaviors e.g., growth, differentiation, metabolism, proptosis, and gaining deeper insights into the molecular mechanisms of complex diseases. While there have been many successes in developing computational approaches for identifying potential genes and proteins involved in cell signaling, new methods are needed for identifying network structures that depict underlying signal cascading mechanisms. In this paper, we propose a new computational approach for inferring signaling network structures from overlapping gene sets related to the networks. In the proposed approach, a signaling network is represented as a directed graph and is viewed as a union of many active paths representing linear and overlapping chains of signal cascading activities in the network. Gene sets represent the sets of genes participating in active paths without prior knowledge of the order in which genes occur within each path. From a compendium of unordered gene sets, the proposed algorithm reconstructs the underlying network structure through evolution of synergistic active paths. In our context, the extent of edge overlapping among active paths is used to define the synergy present in a network. We evaluated the performance of the proposed algorithm in terms of its convergence and recovering true active paths by utilizing four gene set compendiums derived from the KEGG database. Evaluation of results demonstrate the ability of the algorithm in reconstructing the underlying networks with high accuracy and precision.

## Introduction

Inference of signaling networks is critical for deciphering regulatory relationships in living cells and gaining deeper insights into the molecular mechanisms of complex diseases. A signaling network comprises of a complex web of signaling cascades triggered by the binding of external ligands to the transmembrane receptors. Signaling cascades involve a sequential activation of signaling molecules within the cell to lead to a biological end-point function [1]. Computational systems biology approaches serve as a primary mean to understand such complicated wiring of biomolecular interaction and regulation mechanisms. Several approaches have been proposed in the past for inferring these mechanisms including Bayesian networks [2, 3], Boolean or probabilistic Boolean networks [4–6], mutual information networks [7–9], Gaussian graphical models [10, 11] and others [12–16].

One of the earliest network discovery approaches was the so-called relevance networks reconstructed based on pairwise gene expression similarities [17–19]. Commonly used similarity metrics include correlation coefficient [18, 19], partial correlation [10, 17], and mutual information [7, 20]. These approaches permit reconstructing large-scale networks. However, they focus on discovery of local network structures in a pairwise manner, ignoring global, and many-to-many dependencies among genes. Gaussian graphical models and other approaches attempt to infer a global network structure by calculating a full-order partial correlation, i.e., a pairwise feature correlation excluding all other features [10, 11]. However, this approach only discovers one-to-one gene relationships, and the performance is significantly limited for high dimensional data, where the number of genes is larger than the number of samples.

Compared with pairwise similarity based network discovery methods, Bayesian network approaches are more powerful since they consider many-to-one gene dependencies [2, 3, 21]. Numerous strategies for network scoring and searching have been proposed, such as Bayesian Dirichlet (BD) [22], K2 [23] and MCMC [24]. These approaches have stimulated network discoveries across many scientific disciplines. Nevertheless, an important caveat is that the Bayesian networks infer a statistical causal network of genes and not necessarily the physical network structures per se. For high dimensional data (e.g., biological signaling networks with hundreds of genes), network structure discovery using Bayesian network approaches present a computationally daunting task. In order to keep the computation tractable, the size of the parent gene set is often limited to three. Therefore, the reconstructed networks can fail to reveal the genuine many-to-one regulatory relationships. Network reconstruction from gene sets has emerged as an attractive alternative by accommodating many-to-many gene relationships. Note that the number of gene sets is usually much lower than that of the genes due to the overlaps among gene sets. In addition, using gene sets automatically accounts for the many-to-many gene dependency. Recent publications have demonstrated the promising potential of gene set based approaches (e.g., [25–27]). These network discovery approaches take gene sets as the direct structural information emitted from the underlying network, and infer the structure using computational approaches.

There are two major aspects related to a reliable inference of signaling network topologies. First is the identification of the group of molecules involved in a signaling network, and the second aspect is associated with the inference of the network among the molecules involved in signal cascading activities. While there have been many successes in developing computational approaches for identifying potential genes and proteins involved in cell signaling [28, 29], new methods are needed for identifying network structures that depict underlying signal cascading mechanisms. Besides few exceptions [25, 26, 30, 31] most of the existing network inference approaches center around statistical causal interactions and pairwise similarities without explicit consideration of signal cascading activities within their frameworks. Although many annotated signaling pathways and tools for their analysis have become available in recent years [32–37], our current knowledge about signaling mechanisms is still very limited. Existing networks may not necessarily present a complete picture of the the underlying signal cascading activities. Moreover, pathway structures available in public domains are often generic, while scientists may particularly be interested in understanding context-specific signaling networks. Clearly, there is a need for new computational approaches for inferring signaling network structures.

We attempt to address the issues raised above by proposing a new genetic algorithm (GA) [38–40] based approach for inferring signaling network structures from overlapping gene sets related to the networks. The novelty of the proposed approach lies in inferring the underlying signaling network structure through evolution of synergistic subnetworks. We begin with analyzing the structure of a signaling network. A signaling network can be represented as a directed graph and can be viewed as a union of several directed and overlapping chains of signaling cascades, which we refer to as *active paths*. Indeed, active subnetworks have been defined as connected sets of genes with very high differential expression levels [41]. Under the above hypothesis, the true signaling network can be constructed by assembling the active paths into one unit. In other words, active paths can be treated as the basic building blocks of the underlying network. The extent of edge overlapping among active paths facilitates the network construction and can be viewed as *synergy* present among the paths. We propose to infer the underlying active paths and the signaling network from observed gene sets corresponding to active paths. More specifically, gene sets represent the sets of genes participating in active paths without prior knowledge of the order in which genes occur within each path. Thus, an active path and the corresponding gene set carry the same set of genes, however, the directionality information or the arrangement of genes within active paths is unavailable in the case of gene sets. From a compendium of unordered gene sets, GA reconstructs the underlying network structure through evolution of synergistic active paths. In the proposed approach, synergy among active paths is quantified by treating gene sets as random samples from a first order Markov chain model.

The primary motivation for developing a genetic algorithm approach is twofold. First, the exhaustive enumeration of all candidate network structures to locate the true network may be computationally challenging. Indeed, a total of $\prod _{i=1}^{m}L!$ network structures can be constructed from *m* gene sets of lengths *L*, which may be a very large candidate pool even when the values of *m* and *L* are not high. And second, genetic algorithm may be more advantageous compared to previously proposed sampling or search strategies [25, 26] in terms of its ability to avoid being trapped in local solution since it works with a population of solutions at each generation instead of a single solution. As a result, we translate our goal of signaling network inference into a maximization problem and devise a genetic algorithm based search scheme to locate the structure with the maximum synergy among active paths (the true network).

GA is a population based search strategy that utilizes “survival of the fittest mechanism” [38–40]. In the present context, the search space for GA or *feasible set* is defined as the set of all signaling network structures possessing the same degree distribution as the true network, where the true network has the maximum ‘fitness’ (synergy among active paths) in the feasible set. The algorithm starts with an initial population of signaling network structures from the feasible set. In GA, members of the feasible set (candidate signaling networks) are encoded as strings of symbols of equal lengths and are called *chromosomes*. We encode a candidate signaling network by assigning labels to the underlying active paths. GA proceeds iteratively, where a new population is created from the current population through formation of *Mating Pool*, which involves selection of parent chromosomes for creating next generation using *a tournament scheme*, and operations referred to as *cross-over*, where active paths are exchanged between two candidate networks, and *mutation* which involves gene ordering permutation within active paths. At each generation, GA aims to create a population with average fitness value which is higher than the one for the previous population. With evolution of better populations of signaling networks, the proposed GA aims to recover the true network possessing the maximum synergy among subnetworks or maximum fitness score.

We evaluated the performance of GA using four gene set compendiums sampled from four signaling network structures available from the KEGG database [42]. The evaluations were performed in terms of convergence trends, subnetwork evolution, and the ability of genetic algorithm in recovering the underlying active paths and networks. We also compared the performance of genetic algorithm with previously proposed simulated annealing approach [25] due to the similarities in the underlying assumptions in the two approaches. Genetic algorithm demonstrated higher precision and F-score values compared to simulated annealing for the same number of generations or samples used in the two approaches.

## Inference of signaling networks as a maximization problem

We formulate the problem of inferring a signaling network structure from gene sets related to the network as a maximization problem. A gene set is defined as a set of genes participating in a specific active path in the underlying signaling network. We assume a linear arrangement of genes within an active path, whereas the ordering information is assumed to be unavailable in the case of gene sets. Throughout, we denote a gene set by *X*_*i*_ and an active path by (*X*_*i*_,*Θ*_*i*_), where *Θ*_*i*_ represents an instantiation of gene orderings in *X*_*i*_, *i*=1,…,*m*. The length of *X*_*i*_ is defined as the number of genes present in *X*_*i*_ and is denoted by *L*_*i*_. The notations $\overline X$ and $(\overline X, \overline \Theta)$ are used to represent the given gene set compendium and the underlying signaling network structure, respectively, where $\overline X = (X_{1},\ldots,X_{m})$ and $\overline \Theta = (\Theta _{1},\ldots,\Theta _{m})$. A signaling network $(\overline X, \overline \Theta)$ is constructed by assembling the underlying active paths (*X*_*i*_,*Θ*_*i*_), *i*=1,…,*m*.

Since *L*_*i*_! different gene orderings are possible for the gene set *X*_*i*_, a total of $\prod _{i=1}^{m}L_{i}!$ different network structures can be constructed from the gene set compendium $\overline X$. It may be computationally challenging to exhaustively enumerate all $\prod _{i=1}^{m}L_{i}!$ structures and identify the true structure even when the values of *L*_*i*_ and *m* are not large. To address this challenge, we formulate the inference of signaling networks from gene sets as a maximization problem and utilize a search strategy to locate the true structure in the search space, where the true structure receives the highest score among all candidate structures. The maximization problem is formulated as:

(1) $$\begin{array}{@{}rcl@{}} \max_{(\overline X,\overline \Theta) \in \mathcal F_{\overline X}} f(\overline {X},\overline \Theta)  \end{array} $$

where *f* represents the score of a candidate network $(\overline {X},\overline \Theta)$ and $\mathcal F_{\overline X}$ stands for the set of feasible networks. In the next section, we define the search space as well as the scoring function and propose a genetic algorithm (GA) based approach to locate the true network structure.

## A genetic algorithm based search strategy

### The search space for GA

To avoid random networks from consideration, we define the search space or the feasible set for GA using network structures which possess the degree distribution of the underlying network. Since we treat active paths as the basic building blocks of the underlying network, the feasible set can be defined by the networks which are obtained by fixing the pair of terminal genes and permuting the order of intermediate genes in the true active paths. This is because, the incoming and outgoing degrees of the intermediate genes in each active path is 1 which does not get affected even when these nodes are randomly permuted. This results in a feasible set $\mathcal F_{\overline X}$ of size $\prod _{i=1}^{m} (L_{i}-2)!$ for GA where all network structure share the same degree distribution as the true network [25]. Throughout, we refer to the members of a feasible set as “feasible networks”. Biologically, in a signal transduction cascade, terminal genes are easier to determine and are usually available as biological prior knowledge. For instance, the starting node is usually a transmembrane protein, which triggers and transmits signaling cascades. The ending node is usually a transcription factor, which is to turn on/off transcription. Both terminal genes can be recognized by their functional annotations, and use of this prior knowledge can greatly increase the chance of arriving at a global optimal signaling network.

### The representation scheme

We encode each candidate structure in $\mathcal F_{\overline X}$ as a chromosome. The encoding is performed in three steps: (1) enumerating the possible orderings associated with each gene set *X*_*i*_, *i*=1,…,*m* individually, (2) assigning a label to each ordering (active paths), and (3) concatenating the labels of the active paths which define the given signaling network.

### Capturing the synergy among subnetworks

Since we consider a signaling network as a union of active paths, it is necessary to capture the synergy among the active paths in the candidate networks to facilitate the search for the true structure. To achieve this goal, we treat gene sets as random samples from a first order Markov chain model and estimate the two model parameters, initial probability vector *p*_0_ and transition probability matrix *Π*, as $p_{0} = (\frac {c_{1}}{m},\ldots,\frac {c_{n}}{m})$ and *Π*=[*p*_*jk*_]_*n*×*n*_, where *m* is the number of active paths, *n* is the number of distinct genes among the active paths, *c*_*i*_ is the number of times *i* ^*th*^ gene appears as the first node among *m* active paths, *i*=1,…,*n*, $p_{\textit {jk}} = c_{\textit {jk}}/\sum _{k=1}^{n} c_{\textit {jk}}$, *j*,*k*=1,…,*n*, and *c*_*jk*_ is the number of times *j* ^*th*^ gene transits to *k* ^*th*^ gene among *m* active paths. The matrix *Π* captures the edge overlapping information in the given network which defines the synergy among the active paths. The above parameters have also been used in the gene set based approaches proposed in [25, 26].

### Scoring the fitness of a signaling network or the synergy among subnetworks

We utilize the following scoring function to measure the fitness of a candidate signaling network $(\overline X,\overline \Theta)$:

(2) $$\begin{array}{*{20}l} f(\overline X,\overline \Theta)~&=~\log (\mathcal L(\overline X,\overline \Theta))~=~\log\left(\prod_{i=1}^{m} \mathcal \ell(X_{i},\Theta_{i})\right)\\ &=~\sum_{i=1}^{m} \log \mathcal \ell(X_{i},\Theta_{i}),  \end{array} $$

where *ℓ*(*X*_*i*_,*Θ*_*i*_) and $\mathcal L(\overline X,\overline \Theta)$ represent the likelihood of the active path (*X*_*i*_,*Θ*_*i*_) and the signaling network $(\overline X,\overline \Theta)$, respectively, and $\mathcal L(\overline X,\overline \Theta)~=~\prod _{i=1}^{m} \mathcal \ell (X_{i},\Theta _{i})$. The likelihood *ℓ*(*X*_*i*_,*Θ*_*i*_) is calculated using the Markov chain parameters defined above. For instance, the likelihood of an active path $a\rightarrow b \rightarrow c \rightarrow d$ is calculated as *p*_0_(*a*)×*p*_*ab*_×*p*_*bc*_×*p*_*cd*_. From Eq. 2, the problem of searching for the network with the maximum fitness score becomes equivalent to the problem of finding the network with the maximum likelihood in the search space.

### Mating pool

From a given population *P* ^(*k*)^ of chromosomes, we create a mating pool *M* ^(*k*)^ by utilizing a *tournament scheme*. The pool is generated by randomly selecting a pair of chromosomes and placing the chromosome with better fitness value into the pool. If the size of the population is *s*, the tournament is repeated *s* times.

### Cross-over

In cross-over, we randomly select certain pairs of parent chromosomes from the mating pool and exchange a pre-specified number of active paths between them. It is ensured that the active paths which are exchanged between the parents correspond to the same gene sets in the two chromosomes.

### Mutation

The mutation operation is performed by considering each chromosome in *M* ^(*k*)^ and randomly permuting the ordering of intermediate genes in each of the *m* active paths with a very small probability by keeping the terminal genes fixed.

### Elitism

The mating pool *M* ^(*k*)^ obtained after applying cross-over and mutation operations represents the new population or generation *P* ^(*k*+1)^. However, we can further restrict a pre-specified proportion of chromosomes with the highest fitness values in the current population to transfer to the next population without going through cross-over or mutation. We refer to this scheme as elitism.

GA iteratively repeats the above steps until a specified number of generations is reached. This approach has been presented in Algorithm 1.



## Results

### Datasets

We evaluated the performance of GA in inferring the true active paths and networks by utilizing four gene set compendiums derived from four different signaling pathway structures in the KEGG database [42]. The KEGG pathways used in our study are Wnt signaling pathway (hsa04310), axon guidance pathway (hsa04360), leukocyte transendothelial migration pathway (hsa04670), and dilated cardiomyopathy pathway (hsa05414). We utilized the path sampling algorithm proposed in [25] for sampling true active paths from each of the four network structures individually. For deriving gene sets corresponding to the active paths, we randomly permuted the ordering of genes within each active path by keeping the pair of end nodes fixed. This resulted in four gene set compendiums comprising of different numbers and lengths of gene sets which served as input for evaluating the performance of the proposed algorithm. Within each compendium, we only considered gene sets comprising of a minimum of four genes since the gene sets of lengths two or three represent true active paths. We applied GA on each of the four compendiums to infer the true active paths corresponding to the gene sets. The active paths inferred by GA were assembled to reconstruct the underlying subnetwork and network structures. The true subnetworks and networks were constructed by assembling the true active paths. A description of the above datasets is presented in Table 1.

**Table 1:** Description of the datasets

	KEGG Pathway	Number of	Number	Path
		sampled paths	of genes	lengths
Network 1	hsa04310	108	55	min = 4
				max = 7
				mean = 5
Network 2	hsa04360	56	52	min = 4
				max = 7
				mean = 5
Network 3	hsa04670	127	66	min = 4
				max = 8
				mean = 5
Network 4	hsa05414	85	38	min = 4
				max = 7
				mean = 5

### Performance evaluation

#### Fitness of the true signaling networks vs. other feasible networks

We performed an evaluation to show that the true signaling networks have the highest fitness score in the feasible set of network structures by utilizing an empirical statistical test. For each of the four networks, we randomly selected 1000 feasible structures and computed an empirical *P* value *M*/1000, where *M* represents the number of networks with fitness score higher than that of the true structure. We observed that the empirical *P* values corresponding to the true network structures were always zero. We also repeated the above test on four randomly selected feasible networks, one from each of the four search spaces. In this case, the empirical *P* values varied between 0 and 1. This experiment justified the choice of the fitness function used within the proposed algorithm (See Table 2).

**Table 2:** Empirical *P* values of the true signaling networks and other feasible networks

	Empirical *P* value
True network 1	0
True network 2	0
True network 3	0
True network 4	0
Feasible network 1	0.29
Feasible network 2	0.16
Feasible network 3	0.82
Feasible network 4	0.55

#### Convergence performance of GA

**Fig. 1:**
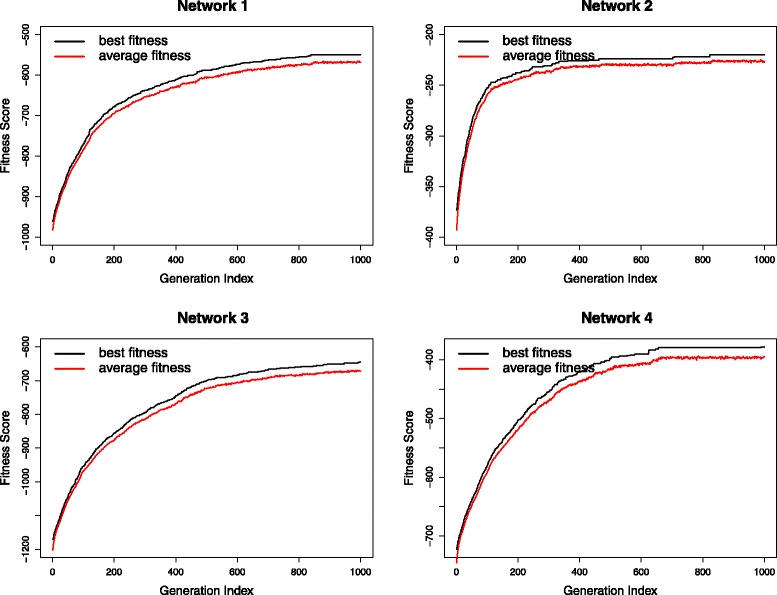
Convergence trend of genetic algorithm for Networks 1−4. Here, the black curve represents the fitness score of the best network discovered at the corresponding generation index, whereas the red curve represents the average fitness score of the population at the corresponding generation index

Using each of the four datasets, we examined the convergence performance of GA in recovering the true network structures. As the current population of chromosomes evolves into a better population within the framework of GA, we expected to observe an increasing trend in the fitness score of the best inferred structure at each generation as well as an increasing trend in the average fitness score of the structures in a population with increasing number of generations. Throughout, we evaluated the performance of GA by fixing the algorithm parameters at *s*=50, *p*_*C*_=0.25, *p*_*M*_=0.01, *p*_*E*_=0.25, *c*_*N*_=1, and *J*=1000. The parameter values were chosen based on the observations from different experiments. For instance, a small population size may not lead to a satisfactory solution while a large population size may increase the computational time. The population size 50 was chosen to achieve a balance between the two factors. The performance of the algorithm was measured in five independent runs. We present the average performance of the algorithm in the figures. In Fig. 1, we present the fitness scores of the best inferred network and the average fitness score of the population at each generation index for the four networks. In each case, we clearly observe an increasing trend in the scores with increase in the number of generations.

**Fig. 2:**
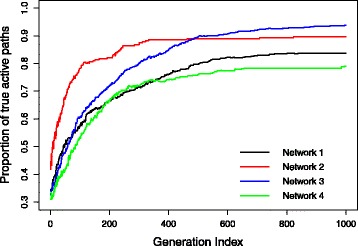
Performance of genetic algorithm in recovering true active paths. Here, each curve corresponds to a specific network and represents the proportion of true active paths inferred by GA at a given generation index. For Networks 1–4, the proportions of true active paths successfully recovered at the end of 1000 generations are 84, 90, 94, and 79 *%*, respectively

We further evaluated the performance of GA in recovering the true active paths with the chosen set of parameters. Figure 2 demonstrates this performance in terms of the proportion of the true active paths in the best inferred network at a given generation index for the four datasets. We observed that >79 *%* of the true active paths are successfully recovered by the algorithm at the end of 1000 generations for each network, whereas the proportions are ≥90 *%* in case of Networks 2 and 3.

#### Synergy among the subnetworks

It is important to note that the proposed algorithm infers the true network structure by considering active paths as the basic building blocks of the network. For recovering the true underlying structure, the ordering of genes within the gene sets in the given compendium is updated over generations through formation of mating pool and operations such as cross-over and mutation. At any given generation, the fitness of a network structure relies on the synergy (overlapping) among the underlying active paths inferred at that stage. With the creation of a new generation, it is possible that certain active paths become more synergistic to each other and result in an overall better network with better fitness value, however, the likelihood scores of some of the underlying active paths and subnetworks formed by these active paths are either increased or decreased. In other words, the likelihood score of a randomly selected subset of active paths, which corresponds to a subnetwork, does not necessarily demonstrate an increasing trend with increasing number of generations as in the case of networks in Fig. 1. As the algorithm approaches towards convergence, the synergy among the active paths increases and in the case of global convergence, the synergy among the active paths is highest in the inferred network, i.e., the fitness of the active paths and hence the subnetworks formed by the active paths is the highest among all generations. In the context of genetic selection for animals resistant to certain diseases, if we treat each of the true active paths as a disease resistant trait and if the selection is performed for animals with these traits, it is not necessary that the selection will lead to perfect population in the next generation.

**Fig. 3:**
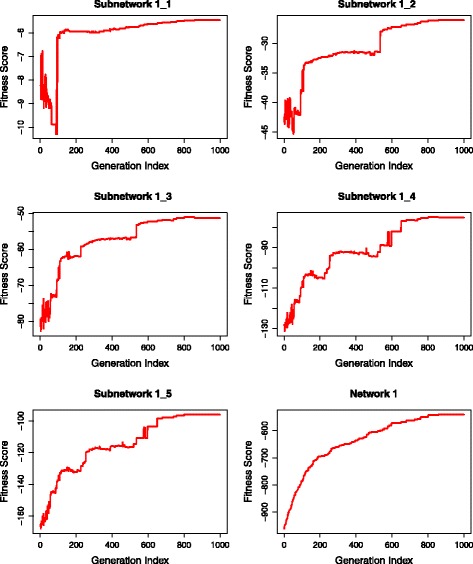
Fitness scores of the structures corresponding to 1, 5, 10, 15, and 20 gene sets (Subplots 1–5) as well as all gene sets corresponding to Network 1 (Subplot 6) over 1000 generations. The best network available at each generation index was used in calculating the fitness scores

We illustrate the above characteristic of GA in Fig. 3. From each of the four datasets, we randomly selected a gene set and tracked the fitness score of the active path formed by the genes in the gene set in the best network available at Generation Index 1,…,1000. For Networks 1−4, we denote the underlying true active paths by Subnetworks *i*_1_, where *i*=1,2,3,4. We further included four randomly selected gene sets from each of the datasets in our experiments and tracked the fitness scores of the subnetworks formed by combining five active paths in the best networks discovered at Generation Index 1,…,1000. In this case, the underlying true subnetworks are denoted by Subnetwork *i*_2_, where *i*=1,2,3,4. We repeated the above procedure to track the fitness of subnetworks formed by 10, 15, and 20 active paths, which we denote by Subnetwork *i*_*j*_, where *j*=3,4,5, and *i*=1,2,3,4. Figure 3, represent the likelihood scores of the above subnetworks of Network 1 over 1000 generations. It is evident from the plots that the discovery of Subnetworks *i*_*j*_, for *i*=1,…,4, *j*=1,…,5, does not necessarily follow the smooth increasing trend in the fitness score (Subplots 1–5) as in the case of Networks 1 (Subplot 6). Different subnetwork structures are explored at each generation to discover the network with an overall better synergy among the active paths.

**Fig. 4:**
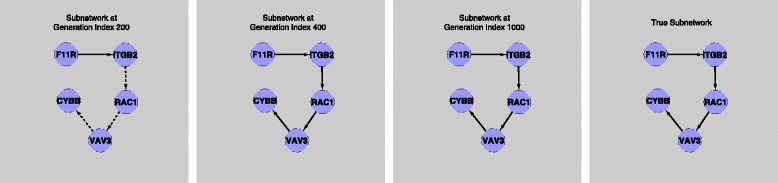
Evolution of a subnetwork corresponding to a randomly selected gene set in the case of Leukocyte transendothelial migration pathway. *Solid edges* with *solid arrows* represent true positives and *dashed edges* with solid arrows correspond to false positives

**Fig. 5:**
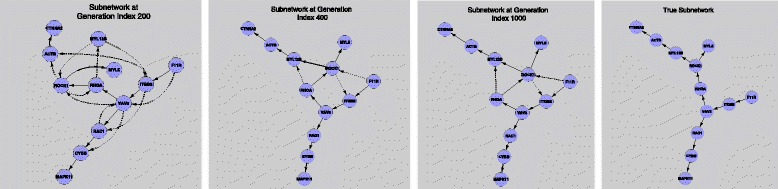
Evolution of a subnetwork corresponding to five gene sets in the case of Leukocyte transendothelial migration pathway. *Solid edges* with *solid arrows* represent true positives and *dashed edges* with *solid arrows* correspond to false positives

**Fig. 6:**
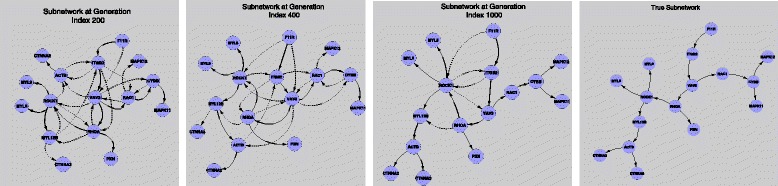
Evolution of a subnetwork corresponding to ten gene sets in the case of Leukocyte transendothelial migration pathway. *Solid edges* with *solid arrows* represent true positives and *dashed edges* with *solid arrows* correspond to false positives

**Fig. 7:**
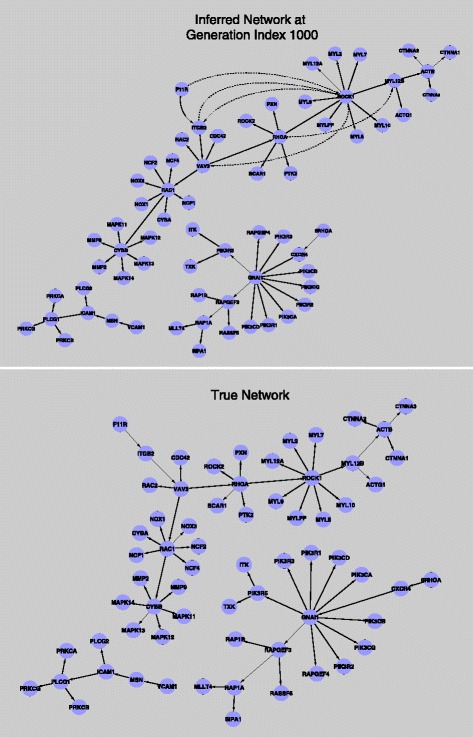
The signaling network structure predicted by GA (*upper*) and the true underlying structure (*lower*) in the case of Leukocyte transendothelial migration pathway. *Solid edges* with *solid arrows* represent true positives and *dashed edges* with *solid arrows* correspond to false positives

In Figs. 4, 5, and 6, we present the evolution of subnetwork structures in Network 3 (Leukocyte transendothelial migration pathway) formed by considering 1, 5, and 10 gene sets, respectively, at different generation indices. Figure 7 represents the true network structure and the structure predicted by GA.

#### Comparison of GA with simulated annealing approach

**Fig. 8:**
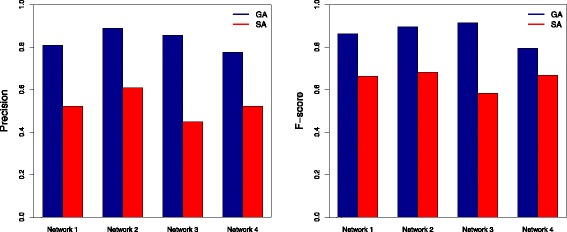
Performance comparison of GA and SA in terms of Precision (*left*) and F-score (*right*)

We compared the performance of GA with the simulated annealing (SA) approach proposed in [25] due to the similarities in the underlying assumptions in the two approaches. The performances were compared in terms of the Precision, which is defined as the proportion of true positives among all predicted edges, and F-score, which is defined as 2*p* *r*/(*p*+*r*), where *p* and *r* represent precision and recall, respectively. The best networks inferred at the end of 1000 generations/samplings were used in the comparison. In the case of SA, the cooling schedule constant was fixed at 10 [25]. Results from these comparisons are presented in Fig. 8. For each of the four networks, we observed a higher F-score and Precision in the case of GA. Note that GA requires more computational time than SA at each iteration since it performs multiple operations to create a new population. On the other hand, SA is based on drawing a random sample and accepting or rejecting it with certain probability which is much faster to perform. However, the results reported here, for an independent run of GA, were obtained in less than 30 min using a standard desktop machine.

## Conclusions

In this paper, we proposed a new genetic algorithm (GA) based approach to reconstruct signaling network structures from gene set compendiums related to the networks. We represented a signaling network structure as a union of overlapping active paths and utilized GA to infer the underlying structure from unordered gene sets corresponding to the paths. The novelty of the proposed approach lies in the inference of the underlying structure through evolution of synergistic subnetworks. In the proposed approach, gene sets were treated as random samples from a first order Markov chain model which allowed us to quantify the synergy among the subnetworks in the evolutionary process. Performance of GA in terms of convergence and recovering the true active paths as well as the network structures was evaluated using four gene set compendiums derived from the KEGG database. Our evaluations demonstrate that GA can predict the underlying network structures with high precision and F-score values. In future studies, the proposed method can be integrated with the approaches for discovering pathways from big molecular profiling datasets to derive novel signaling network topologies and constructing context-specific signaling networks.
